# Does a low-carbohydrate diet impede endurance sports performance? No

**DOI:** 10.1016/j.ajcnut.2026.101269

**Published:** 2026-05-01

**Authors:** Timothy D Noakes, Louise M Burke

**Affiliations:** 1 Emeritus Professor, University of Cape Town, Cape Town, South Africa; 2 Mary MacKillop Institute for Health Research, Australian Catholic University, Melbourne, Victoria, Australia

**Keywords:** low-carbohydrate diet, exercise performance, small glucose pool, large glucose pool, exercise-induced hypoglycemia, muscle glycogen, fat oxidation

## Abstract

Over the past decade, randomized controlled trials have established that, after eating either a low-carbohydrate, high-fat or a high-carbohydrate, low-fat diet for 4–6 wk, trained athletes performed equally well during a maximum oxygen consumption (VO_2_max) test; during 5 and 1.6 km laboratory treadmill time trials; during a 6 × 800 m interval repetition session; and during a prolonged cycling test to exhaustion at 70%VO_2_max. Indeed, during the 6 × 800 m interval repetition session, some subjects achieved the highest rates of fat oxidation (2 g/min) ever reported in humans; whereas ingestion of 10 g carbohydrate/h improved prolonged cycling test performance by 22%, equally following either diet. These data establish that muscle glycogen is not an obligatory fuel for exercise. Rather, exercise-induced hypoglycemia due to depletion of glucose in the small glucose pool in the liver and bloodstream, prevented by minimal carbohydrate ingestion during exercise, is the main metabolic contributor to premature fatigue during more prolonged submaximal exercise.

## Main Argument (Noakes)

For over 35 y, I promoted a high-carbohydrate (CHO) low-fat diet (HCLF) to optimize health and athletic performance. This included statements [1] asserting that diets high in CHO are essential for training and competition, primarily because of a perceived critical role for muscle glycogen in fueling both high-intensity [>85% maximum oxygen consumption (VO_2_max)] [2,3] and prolonged exercise [[2], [3], [4], [5], [6], [7]]. I considered this to be “settled science.”

Since December 2010, my certainty has unraveled. Utilizing randomized controlled trials (RCTs), our research has systematically compared HCLF and low-CHO high-fat (LCHF) diets across various exercise performance tests including a VO_2_max test [8], 5 km [8], and 1.6 km [9] treadmill time trials, a treadmill running session in which subjects ran 800 m at high intensity followed by a period of rest repeated 6 times [9], a time-to-fatigue trial at 70% VO_2_max [10], and an analysis of over 500 studies published over the past century [11]. This evidence made me question the essential role of muscle glycogen in determining human athletic performance. Challenging orthodox nutritional doctrines triggered backlash [12]. However, rigorous science demands adherence to evidence over conventional orthodoxy.

This work established that eating a LCHF diet for 4–6 wk increased the exercise intensity threshold (from 50% to 85%VO_2_max) at which the transition from predominantly fat to CHO oxidation occurs [13,14], indicating improved metabolic flexibility. Six times 800 m intervals run at 86% VO_2_max, produced the highest rate of fat oxidation (>2.1 g/min (Figure 5 in [9]) ever measured in an exercising human.

These studies disprove the theory that only high rates of CHO oxidation [2,3,5,15,16] can sustain exercise at >85% VO_2_max. The method of measurement, respiratory gas analysis [6,17], also overestimates the true extent of CHO oxidation as exhaled CO_2_ arises from body CO_2_ stores, not from muscle metabolism [18], artificially inflating the apparent contribution of CHO oxidation at higher exercise intensities.

Our most important study [10] evaluated metabolic and performance effects of ingesting either a placebo or a low-CHO dose during prolonged-strenuous exercise at 70% VO_2_max after chronic (6 wk) exposure to either the LCHF or HCLF diets. This RCT tested the relative performance effects of diet-induced changes in the small glucose pool (SGP) in the liver and bloodstream or of skeletal muscle glycogen in the large glucose pool (LGP) [11]. The SGP and LGP are differentially regulated [11] as *1)* ingested or infused CHO increases pancreatic insulin secretion, reducing liver glycogenolysis, sparing CHO in the SGP but, *2)* by reducing adipose tissue lipolysis and, as a result, skeletal muscle fat oxidation, CHO ingestion or infusion directly increases CHO use from the LGP [19,20]; *3)* this effect may be regulated in a dose-dependent manner by blood glucose concentrations, as infusion-induced hyperglycemia exaggerates these effects [19].

Accordingly, we administered minimal CHO supplementation (10 g/h) to prevent exercise-induced hypoglycemia without substantially impacting LGP CHO metabolism. In an RCT that manipulated the respective CHO contents of the LGP through habitual diet and pre-exercise fasting, and that of the SGP by CHO supplementation during exercise, we tested whether prolonged exercise performance was more strongly influenced by *1)* habitual long-term provision of dietary CHO before exercise to fill the LGP [2,3] and prolong submaximal exercise performance [[2], [3], [4], [5], [6], [7],^10^,^21^] or *2)* short-term provision of CHO to the SGP during exercise [11].

Our findings were unequivocal: LGP manipulation did not influence exercise performance confirming our previous finding [8,9]. However, maintaining the glucose content of the SGP to prevent exercise-induced hypoglycemia, increased exercise time to fatigue to an equal extent (12%–22%) after both the LCHF and HCLF dietary interventions.

This finding confirms my original argument [22] that Christensen and Hansen [23] were correct when they concluded in 1939 that: “…the beneficial effect [of CHO ingestion during exercise) is only present if this carbohydrate supply forces the blood sugar concentration to rise [so that] … Fatigue must be regarded as a hypoglycemic symptom of cerebral origin” (p. 178).

At least 3 of the most popular doctrines supporting claims for the superiority of HCLF diet are false:

1) *Human skeletal muscle cannot extract sufficient energy from fat oxidation once the exercise intensity exceeds 85%VO*_*2*_*max (Figures 1 and 2 in* [15] *and in* [16]*), explaining why the LCHF diet impairs performance during high-intensity exercise.* Instead, during high-intensity exercise [9], we measured rates of fat oxidation that could provide >90% of the energy required by a 52 kg athlete to run a sub-2 h marathon [22]. This suggests that muscle glycogen is not the obligatory fuel for skeletal muscle metabolism, at least up to an exercise intensity of 85%VO_2_max. Our finding that performances during 5 [1], [8].6 km time trials [9], during the VO_2_max test [8] and during 6 × 800 m interval repetitions [9] were not enhanced by a diet designed to increase the LGP, supports this interpretation.
2) *LGP is the primary determinant of performance during prolonged exercise.* Our findings that prolonged exercise performance was improved by the manipulation of the CHO content of SGP during exercise thereby preventing exercise-induced hypoglycemia, but not by dietary interventions aimed at altering the CHO content of the LGP before exercise, establish that the SGP, not the LGP, is the more important factor determining the (initial) fatigue that develops during prolonged exercise. Importantly, the prevention of exercise-induced hypoglycemia will not allow the exercise to continue indefinitely [24], indicating that other, presumably nonmetabolic factors, become the more important determinants of fatigue during prolonged exercise, lasting more than 3–4 h.
3) *Exogenous CHO ingested at a very high rate (30–90 g/h)* [21] *is required to sustain prolonged exercise performance.* Once the “obligatory” muscle glycogen content of the LGP is depleted, it is unnecessary to provide exogenous CHO at a very high rate (30–90 g/h) [21] to sustain the exercise, since exercise-induced hypoglycemia, due to depletion of the SGP rather than muscle glycogen depletion in the LGP, is the cause of this brain-directed fatigue [11,23]. Prevention of exercise-induced hypoglycemia during prolonged exercise requires the ingestion of CHO at relatively low rates (∼10 g/h) since the SGP contributes a relatively small fraction of total CHO oxidation during exercise [11].

These points support a large body of RCT and other evidence, as previously argued [22,23] that habitual consumption of a LCHF diet does not impair exercise performance after an adequate period of physiological adaptation (≥4 wk). Indeed, a diet specifically designed to increase the pre-exercise glycogen content of the LGP [21] consistently fails to enhance performance in a wide range of different athletic activities.

Thus systematic reviews of 17 [25] and 13 [26] studies concluded that “physical performance can be maintained when consuming a [very-low-carbohydrate] ketogenic diet compared with control [25],” and that an athlete involved in “moderate-to-vigorous intensity exercise experiences no decrement (in performance) following adaptation to a ketogenic diet” [26]. Another meta-analysis of 10 studies found that the ketogenic diet “did not affect aerobic capacity and endurance performance” [27]. After adaptation to the ketogenic diet, “endurance-trained athletes experienced no decrement in endurance capacity” during exercise of either moderate (46%–63% VO_2_max) or vigorous intensity(64%–90%VO_2_max) [26]. Trained gymnasts and taekwondo athletes experienced “no decrement in strength endurance and power, and isometric strength,” whereas resistance-trained athletes “maintained 1RM back squat, bench press, clean, jerk and deadlift performance.” Performances over 2000-m, 400-m, 100-m, 6-s sprints, 5 3-min interval sprints, and Wingate testing were also unimpaired after adoption of the ketogenic diet [26]. Professional firefighters who followed a CHO-restricted diet for 28 d lost weight, reduced their blood pressures and improved performance on the firefighters’ performance assessment (2.41 km run time; pull-up performance) without changes in Wingate test results [28]. The authors concluded that “a 28-day carbohydrate-restricted diet can benefit markers of health in professional firefighters without detriment to occupational performance.”

Another 3 RCTs found that the low-CHO diet did not impair running performance during VO_2_max testing, during a 20-min running time trial or during a 21 km running race [29]; during VO_2_max testing or during a run to exhaustion at 70%VO_2_max in which subjects ingested CHO (60 g/h) that prevented exercise-induced hypoglycemia in all groups, in which subjects ran an average of 50 km, and in which terminal rates of CHO and fat oxidation were remarkably different (3.1 compared with 1.2 g CHO/min; 0.4 compared with 1.2 g fat/min: normal compared with low-CHO diet) [30]; or during a 5-h cycling time trial [31].

We have recently completed an extensive analysis of essentially all the trials of the effects on athletic performance of CHO ingestion before exercise (to increase the CHO content of the LGP) or during exercise (to maintain the glucose content of the SGP) [11]. This revealed 16 lines of evidence confirming that the CHO content of the SGP and not that of the LGP (i.e., muscle glycogen) is the factor that influences human exercise performance. Among the most salient points are the following:

*The Anaplerotic Theory (used as the sole explanation of how muscle glycogen depletion causes exercise fatigue) is biologically implausible and disproven*. ATP, not glycogen, is the immediate “fuel” for exercise performance. If muscle ATP concentrations are not maintained during exercise, the result will be skeletal muscle rigor [32,33], not fatigue. There is still no credible, experimentally proven explanation of how muscle glycogen depletion causes the development of a progressive, apparently regulated fatigue. Instead, researchers quote associational studies in which muscle glycogen content falls as fatigue develops [34,35], as evidence for an (assumed) causal relationship. However, association does not prove causation.

*The original Scandinavian study* [4] *linking muscle glycogen and exercise duration ignored key findings of hypoglycemia and high rates of fat oxidation in CHO-restricted subjects.* Importantly, this original study was observational, precluding causal inference. The sole metabolic similarity in all groups at exhaustion was low blood glucose concentration, suggesting that this critical metabolic parameter is the most likely determinant of exercise termination [11,22].

*In studies showing CHO improved performance, blood glucose concentrations decreased progressively during exercise in ≤88% of placebo groups*. Whereas in studies in which exercise performance was not improved by CHO ingestion, blood glucose concentrations were reduced in only 30% of the control groups. CHO ingestion during exercise improved performance ∼2.7 times more often in studies in which blood glucose concentrations fell in control groups. This is the first review that analyzed the potential effect of blood glucose changes in the control group during exercise on the measured performance outcomes. Until now, this possibility has been overlooked.

*During prolonged exercise, whole-body CHO oxidation declines, whereas blood glucose* o*xidation increases, independent of exercise intensity or the CHO content of the pre-exercise diet*. Exercise-induced hypoglycemia is likely without CHO intake—especially with low liver glycogen and limited gluconeogenesis. This feature of human biology makes hypoglycemia inevitable during prolonged exercise in susceptible individuals if CHO is not ingested. It is as if the human body is designed to deplete the SGP terminating exercise by central brain mechanisms [11,[36], [37], [38]] to avoid other bodily harms.

*Preclinical mechanistic studies suggest that hypoglycemia, rather than muscle glycogen depletion, is the primary “exercise stopper” during prolonged exercise*. This evidence shows that rats with superior capacity to increase the SGP have increased endurance performance, whereas those with increased LGP do not.

*Maximal fat oxidation rates in high-CHO–adapted athletes underestimate fat’s contribution to energy metabolism, even during high-intensity exercise*. The result is that fat oxidation can potentially provide almost all the energy required to run a sub-2 h marathon [23].

*Endurance performance after 6 wk of high- or low-CHO diets showed no difference,* but 10 gCHO/h during exercise on both diets boosted performance by 12%–20%, linked to elimination of hypoglycemia [10].

The evidence is incontrovertible. Although there is no evidence that the low-CHO/ketogenic diet improves athletic performance, there is equally no evidence that, when athletes undergo an appropriate period of dietary adaptation—for example, ≥4 wk [39]—a low-CHO ketogenic diet impairs any type of physical activity that has been studied. A more appropriate conclusion would be that the macronutrient content of the habitual pre-exercise diet consumed for >4–6 wk has no influence on subsequent exercise performance.

## Refutation (Burke)

“Black and White thinking,” “one size fits all,” and “reductionist” approaches were all identified in my Main Argument as a detriment and distraction to insights into sports performance [40]. It is disappointing to applied sports scientists, elite athletes and their embedded teams that despite collaborations and improved scientific understanding of the uniqueness and diversity of performance optimization, others dismiss the nuances and complexity of the endurance sport landscape. Table 1 [2,10,[41], [42], [43], [44], [45], [46], [47]] briefly summarizes each of the components raised in argument that the ketogenic LCHF diet does not impede endurance performance, with evidence that each fails to represent the entirety or the intricacy of high-performance sport. Unfortunately, equal time could also be devoted to the frailty of the research process and its legacy in the sports nutrition literature which has led to some of these (and other) misunderstandings. Limitations in study design or experimental protocols, poor understanding of the basic principles of sports performance or human metabolism, measurement error and imprecision, blind trust in technology over sense checking data and its analysis, and a lack of expertise in applied sports science each takes a toll. These problems are amplified by the crisis in the peer review process, the scourge of predatory journals, and the social media megaphone. Unfortunately, these endemic problems are much harder to solve than the current debate question, but must be woven into any scientific discussion.

**TABLE 1:** Key points in the refutation.

Issues	Explanation of the overall effect on endurance performance
Lack of importance of muscle glycogen depletion as the cause of fatigue/performance decrement.	Current understanding of sports performance does not identify muscle glycogen depletion as the sole determinant with a single causative mechanism [2,41]. According to the specific scenario, glycogen depletion might impair performance via direct (e.g., subcellular changes in excitation-contraction coupling including altered muscle excitability and calcium kinetics) or indirect mechanisms (e.g., changes in economy of ATP production due to reduced CHO contribution) [42]. In other scenarios, it may not contribute to changes in performance: CHO mouth rinsing and CHO intake protocols can enhance performance without changing muscle glycogen use.
The importance of hypoglycemia/low blood glucose as the determinant of fatigue.	Experimental data show many examples in which low blood glucose or changes in blood glucose are not a determinant or sole determinant of sports performance. Rebound hypoglycemia due to pre-exercise feeding shows dissociation between the reduction/absolute blood glucose concentration, subjective measures of fatigue, and exercise performance [43]. Meanwhile, CHO mouth rinsing studies show benefits to performance of selected sporting protocols without changing blood glucose concentrations [44]. Although finding that a CHO supply of 10 g/h can improve endurance in fat-adapted or carbohydrate-supported cohorts is interesting [10], failure to test it against models involving multiple strategies to increase CHO availability [2,41] precludes translation of this strategy to optimization of performance. Meta-analyses of studies of within-exercise CHO intake show benefits to endurance performance, including different mechanisms according to scenario, and evidence for a dose-response ( ≤90 g/h) and an increase in the magnitude of benefit as the duration of exercise increases [45].
Misunderstanding that maximal fat oxidation rates from LCHF adaptation cannot supply all the energy for high-intensity exercise.	Our measurements in elite athletes under steady state conditions confirm that adaptation to a ketogenic LCHF achieves extraordinarily high rates of fat oxidation (e.g., ∼2 g/min in individuals) during sustained high-intensity performance [46]. We agree that almost all the energy to fuel key endurance events could be provided by fat oxidation under such conditions. However, theoretical and empirical data show that for the same oxygen supply, fat oxidation achieves a lower energy yield than exercise supported by carbohydrate oxidation, producing lower power/speed [2,46,47]. This explains the remarkably consistent impairments of real-world race performance in elite athletes associated with after an LCHF intervention, which are apparently even greater in the world-class competitors (Olympic/World Championship medallists) within the cohort [46].
It does not matter whether athletes follow a high CHO or LCHF diet in terms of endurance performance.	High-performance sport is an ecosystem of extremely specific scenarios in which a variety of factors may interact to create differences in the response to any intervention, and in which athletes should be encouraged to understand and address their specific needs. Rather than a blunt meta-analytical approach, our LCHF sports dashboard [47] showcases specific study characteristics to identify scenarios in which a true performance effect might be expected. Current insights include characteristics of the sporting event that disfavor the use of a ketogenic LCHF approach (e.g., sustained high intensity >85% VO_2_max) as well as methodological confounders (e.g., substantial weight loss, order effect, large numbers of dropouts) that might create artifacts in the interpretation of some studies.

Abbreviations: CHO, carbohydrate; LCHF, low-carbohydrate high-fat; VO_2_max, maximum oxygen consumption.

Issues related to the measurement of substrate metabolism during exercise undoubtedly cloud the picture. However, because the chief interest of this series is *performance*, my focus must be on the potential of the literature we have both reviewed to understand and measure this. Metrics of exercise capacity or other isolated aspects (e.g., 1RM strength, Wingate tests, graded exercise tests) may be related to endurance performance but generally fail to provide an adequate proxy for sports performance. Furthermore, an isolated strategy that delays the termination of an exercise task may not represent optimal competition support. Unfortunately, many other methodological aspects of commonly cited papers in the LCHF literature (e.g., participant characteristics, inclusion of distracting tasks or equipment during the “performance” protocol, laboratory conditions, background nutritional characteristics) prevent the studies from capturing valid and reliable measurements of competition performance. Even without examining whether animal studies can truly measure *sports* performance, preclinical models of exercise metabolism do not translate well to humans due to differences in the relative importance of bloodborne (rodents) compared with endogenous (human) fuels [48]. This literature must also be faulted for the general lack of appreciation of the precision needed to detect performance changes of real-world significance or to sense check the ecological validity of the collected data. The assembly and integration of the suite of performance nutrition strategies [41] within the design of most studies are sadly neglected. Meanwhile, the key elements of the participants’ motivation and incentive to perform, particularly within the environment of competition (including interactions with other competitors or spectators), are rarely considered. Special research environments and protocols are needed to address true measurement of sports performance in high-performance athletes [49].

Understanding and working within the elite level of sport demands specialized expertise that recognizes the complexity of human performance, as well as the specificity of the unique demands of the event and the unique characteristics of each athlete. I make no apology for demanding that the bar of the current debate be raised beyond “nothing really matters.” If the intention is to disprove the existence of an effect or the subtlety of specific effects, focusing on the average outcome of a group of heterogeneous interventions is a convenient tool. For example, if each athlete who won a gold medal at the Paris 2024 Olympic or Paralympic Games provided details of the specific training program that led to the victory dais, the expected diversity across sports, events, and individuals would undoubtedly create a global impression that “so much difference must mean that nothing is important.” However, in each individual experience, the detail and its integration to produce the moment are exquisite. How sports nutrition plans are assembled and implemented to support optimal biomechanical, metabolic, psychological, technical, and tactical outcomes does matter. Furthermore, they are likely to involve a combination of strategies targeting various performance limiters, sometimes with redundancy, and suited to the logistics of the event and the individual athlete’s experience of responsiveness [2,41].

I would emphasize the findings from the series of studies from my own research group (highly controlled interventions involving elite athletes, real-world competition, standardization of the belief effect); they show robust evidence, at least in the endurance performance scenarios in which they were set, the LCHF diet is associated with a suboptimal outcome compared with a personalized and integrated suite of strategies that include promotion of high-CHO availability [46,47]. The consistency of our findings, the controlled and ecologically valid setting in which they were produced, and the sound underpinning biochemistry invalidates any statement that the background diet does not matter. Furthermore, I can explain my motivation to pursue decades of research into numerous iterations of “fat adaptation” strategies, including the more recent LCHF interest [46,47], by the desire to thoroughly understand the scenarios in which they might or might not “work.” Because of this, and occasional opportunities to work with other athletes and other scenarios in which an LCHF diet can be a successful strategy, I appreciate that sport is a varied tapestry that deserves respect for its complexity, continuing evolution, and capacity to challenge knowledge and practice. Within this framework, although tinkering with the LCHF diet can remove/reduce some of the characteristics that limit specific aspects of performance, on some occasions at least, it fails to represent the optimal plan [46,47].

## Rebuttal (Noakes)

Professor Burke’s argument is that the findings from her studies are definitive whereas those questioning her conclusions “dismiss the nuances and complexity of the endurance sport landscape”; they “fails to represent the entirety or the intricacy of high-performance sport,” all of which is “disappointing to applied sports scientists, elite athletes and their embedded teams.” This is compounded by “the frailty of the research process and its legacy in the sports nutrition literature.” But these statements are flagrant red herrings; this debate is not about those issues. It is solely about whether the habitual diet alone influences something as mundane and readily measurable as exercise performance.

I present strong evidence from carefully conducted RCTs establishing that the exercise performance of “regular” athletes adapted alternatively to either high- or low-CHO diets for an appropriate duration of ≥4, or preferably 6 wk or longer, is identical on both diets. To counter this, Burke’s argument is the following: Because her studies show that elite athletes perform worse on a low- compared with high-CHO diet, then divergent findings from studies of regular athletes cannot be correct. Rather, these experiments must be flawed and low-CHO diets cannot be promoted for any athletes, elite or regular.

Furthermore, Burke’s studies are not flawless. The perhaps understandable flaws in her Super Nova studies were caused by the complexities inherent in studies of elite athletes. First, her trials were not RCTs. Why would elite athletes preparing for a new competitive season wish to risk randomization to an untested diet, especially one that the senior researcher had herself repeatedly characterized in public and in the published literature as inferior? Second, the duration of the trial (3.5 wk) was set by the athletes’ collective patience, not by a predetermined duration required for the necessary biological adaptations to this novel diet. Then, as now, Burke lacks any certainty of the optimum period of adaptation to the low-CHO diet. Indeed, the exercise performance of the low-CHO group in her latest study (Figure 2C in Ref. [50]) was still improving when the study terminated after 3 wk.

Before embarking on these studies, Burke should *1)* have determined the time course of the performance adaptation to the low-CHO diet; armed with that knowledge, she should then *2)* have recruited only those athletes prepared to be randomly assigned into a dietary RCT of the required duration. Therefore, her studies are best characterized as preliminary investigations of the time course of the initial, early adaptations of elite athletes to a low-CHO diet, rather than as definitive studies proving long-term impaired performance on that diet. Regarding her remaining arguments, I encourage readers to consult our review [11], which details the 16 lines of evidence explaining why the macronutrient composition of the pre-exercise diet, either high or low in CHO, has not been shown to influence exercise performance.

## Disclaimer

This article series is designed as an Oxford-style debate. As such, participants are required to argue pro- and con- positions, even when that opinion may differ from their own. The views expressed in this debate do not necessarily reflect the opinion of the participants, *The American Journal of Clinical Nutrition*, or the American Society for Nutrition.

## Declaration of Generative AI and AI-assisted technologies in the writing process

No AI tools were used in the writing of this manuscript.

## Conflict of interest

TDN is the author of low-carbohydrate nutrition books and a shareholder in The Nutrition Network. All proceeds from these activites are donated to the NGO, The Noakes Foundation. LMB has been an unpaid and short-term member of several Scientific Advisory Boards for Sports Nutrition Companies during selected periods of her career in sports nutrition: Gatorade Sports Science Institute Expert panel and Science in Sports. LMB has received travel grants from organizations related to both the carbohydrate and low-carbohydrate high-fat (LCHF) industries to present on this topic. LMB has worked as a sports dietitian in elite sport for 45 y, supporting high-performance athletes to implement bespoke nutrition plans for their specific sporting pursuits. This includes assisting athletes to adopt periodized approaches to CHO availability as well as specialized use of the LCHF diet for ultraendurance events. LMB has been an unpaid and short-term member of several Scientific Advisory Boards for Sports Nutrition Companies during selected periods of her career in sports nutrition: Gatorade Sports Science Institute Expert panel and Science in Sports.

## References

[1] Noakes T.D. (2003).

[2] Burke L.M., Hawley J.A. (2018). Swifter, higher, stronger: what’s on the menu?. Science.

[3] Hargreaves M., Spriet L.L. (2020). Skeletal muscle energy metabolism during exercise. Nat. Metab..

[4] Bergström J.B., Hermansen L., Hultman E., Saltin B. (1967). Diet, muscle glycogen and physical performance. Acta Physiol. Scand..

[5] Hawley J.A., Leckey J.J. (2015). Carbohydrate dependence during prolonged, intense endurance exercise. Sports Med.

[6] Romijn J.A., Coyle E.F., Sidossis L.S., Gastaldelli A., Horowitz J.F., Endert E. (1993). Regulation of endogenous fat and carbohydrate metabolism in relation to exercise intensity and duration. Am. J. Physiol..

[7] Sherman W.M. (1987). Carbohydrate, muscle glycogen, and improved performance. Phys. Sportsmed..

[8] Prins P.J., Noakes T.D., Welton G.L., Haley S.J., Esbenshade N.J., Atwell A.D. (2019). High rates of fat oxidation induced by a low-carbohydrate, high-fat diet, do not impair 5-km running performance in competitive recreational athletes. J. Sports Sci. Med..

[9] Prins P.J., Noakes T.D., Buga A., D'Agostino D.P., Volek J.S., Buxton J.D. (2023). Low and high carbohydrate isocaloric diets on performance, fat oxidation, glucose and cardiometabolic health in middle age males. Front. Nutr..

[10] Prins P.J., Noakes T.D., Buga A., Gerhart H.D., Cobb B.M., D’Agostino D.P. (2025). Carbohydrate ingestion eliminates hypoglycemia and improves endurance exercise performance in triathletes adapted to very low and high carbohydrate isocaloric diets. Am. J. Physiol. Cell Physiol..

[11] Noakes T.D., Prins P.J., Buga A., D'Agostino D.P., Volek J.S., Koutnik A.P. (2026). Carbohydrate ingestion on exercise metabolism and physical performance. Endocr. Rev..

[12] Noakes T.D., Sboros M. (2019).

[13] Prins P.J., Noakes T.D., Buxton J.D., Welton G.L., Raabe A.S., Scott K.E. (2022). High fat diet improves metabolic flexibility during progressive exercise to exhaustion (VO_2_ max testing) and during 5 km running time trials. Biol. Sport..

[14] Noakes T.D., Prins P., Volek J.S., D’Agostino D.P., Koutnik A.P. (2023). Low carbohydrate high fat ketogenic diets on the exercise crossover point and glucose homeostasis. Front. Physiol..

[15] Achten J., Jeukendrup A.E. (2003). Maximal fat oxidation during exercise in trained men. Int. J. Sports Med..

[16] Achten J., Gleeson M., Jeukendrup A.E. (2002). Determination of the exercise intensity that elicits maximal fat oxidation. Med. Sci. Sports Exerc..

[17] Romijn J., Coyle E., Hibbert J., Wolfe R. (1992). Comparison of indirect calorimetry and a new breath 13C/12C ratio method during strenuous exercise. Am. J. Physiol. Endocrinol. Metab..

[18] Jones N.L., Jurkowski J.E. (1979). Body carbon dioxide storage capacity in exercise. J. Appl. Physiol. Respir. Environ. Exerc. Physiol..

[19] Hawley J.A., Bosch A.N., Weltan S.M., Dennis S.C., Noakes T.D. (1994). Glucose kinetics during prolonged exercise in euglycaemic and hyperglycaemic subjects. Pflugers Arch..

[20] Hawley J.A., Bosch A.N., Weltan S.M., Dennis S.C., Noakes T.D. (1994). Effects of glucose ingestion or glucose infusion on fuel substrate kinetics during prolonged exercise. Eur. J. Appl. Physiol. Occup. Physiol..

[21] Burke L.M., Castell L.M., Casa D.J., Close G.L., Costa R.J., Desbrow B. (2019). International Association of Athletics Federations consensus statement 2019: nutrition for athletics. Int. J. Sport Nutr. Exerc. Metab..

[22] Noakes T.D. (2024). Ketogenic diets are beneficial for athletic performance. Med. Sci. Sports Exerc..

[23] Noakes T.D. (2024). Ketogenic diets are beneficial for athletic performance: response to Burke and Whitfield. Med. Sci. Sports Exerc..

[24] Coyle E.F., Coggan A.R., Hemmert M.K., Ivy J.L. (1986). Muscle glycogen utilization during prolonged strenuous exercise when fed carbohydrate. J. Appl. Physiol..

[25] Murphy N.E., Carrigan C.T., Margolis L.M. (2021). High-fat ketogenic diets and physical performance: a systematic review. Adv. Nutr..

[26] McSwiney F.T., Doyle L., Plews D.J., Zinn C. (2019). Impact of ketogenic diet on athletes: current insights, Open Access J. Sports Med.

[27] Cao J., Lei S., Wang X., Cheng S. (2021). The effect of a ketogenic low-carbohydrate, high-fat diet on aerobic capacity and exercise performance in endurance athletes: a systematic review and meta-analysis. Nutrients.

[28] Waldman H.S., Smith J.W., Lamberth J., Fountain B.J., McAllister M.J. (2019). A 28-day carbohydrate-restricted diet improves markers of cardiometabolic health and performance in professional firefighters, J. Strength Cond. Res..

[29] Vogt M., Puntschart A., Howald H., Mueller B., Mannhart C., Gfeller-Tuescher L. (2003). Effects of dietary fat on muscle substrates, metabolism, and performance in athletes. Med. Sci. Sports Exerc..

[30] Shaw D.M., Merien F., Braakhuis A., Maunder E., Dulson D.K. (2019). Effect of a ketogenic diet on submaximal exercise capacity and efficiency in runners. Med. Sci. Sports Exerc..

[31] O’Connor W., O’Connor E., Barnes M., Miller M., Gardener H., Stannard S. (2022). Chronic carbohydrate restriction does not reduce endurance capacity in men and women. J. Sci. Cycl..

[32] Noakes T.D. (2000). Physiological models to understand exercise fatigue and the adaptations that predict or enhance athletic performance. Scand. J. Med. Sci. Sports..

[33] Noakes T.D., St Clair Gibson A. (2004). Logical limitations to the “catastrophe” models of fatigue during exercise in humans. Br. J. Sports Med..

[34] Jensen R., Ortenblad N., Strausholm M.L.H., Skjaerbaek M.C., Larsen D.N., Hansen M. (2020). Heterogeneity of subcellular muscle glycogen utilization during exercise impacts endurance capacity in men. J. Physiol..

[35] Nielsen J., Jensen R., Ortenblad N. (2024). Assessments of individual fiber glycogen and mitoochondrial volume percentages reveal a graded reduction in muscle oxidative power during prolonged exhaustive exercise. Scand. J. Med. Sci. Sport..

[36] Nybo L., Moller K., Pedersen B., Nielsen B., Secher N. (2003). Association between fatigue and failure to preserve cerebral energy turnover during prolonged exercise. Acta Physiol. Scand..

[37] Nybo L. (2003). CNS fatigue and prolonged exercise: effect of glucose supplementation. Med. Sci. Sports Exerc..

[38] Nybo l., Secher N.H. (2004). Cerebral pertubations provoked by prolonged exercise. Prog. Neurobiol..

[39] Ludwig D.S., Willett W.C., Putt M.E. (2025). Wash-in and washout effects: mitigating bias in short term dietary and other trials. BMJ.

[40] Burke L.M., Noakes T.D. (2026). Does a low-carbohydrate diet impede endurance sports performance? Yes. Am. J. Clin. Nutr..

[41] Burke L.M. (2021). Nutritional approaches to counter performance constraints in high-level sports competition. Exp. Physiol..

[42] Vigh-Larsen J.F., Ortenblad N., Spriet L.L., Overgaard K., Mohr M. (2021). Muscle glycogen metabolism and high-intensity exercise performance: a narrative review. Sports Med.

[43] Jeukendrup A.E., Killer S.C. (2010). The myths surrounding pre-exercise carbohydrate feeding. Ann. Nutr. Metab..

[44] Painelli V.S., Brietzke C., Franco-Alvarenga P.E., Canestri R., Vinícius Í., Pires F.O. (2022). A narrative review of current concerns and future perspectives of the carbohydrate mouth rinse effects on exercise performance. SAGE Open Med.

[45] Stellingwerff T., Cox G.R. (2014). Systematic review: carbohydrate supplementation on exercise performance or capacity of varying durations. Appl. Physiol. Nutr. Metab..

[46] Burke L.M., Whitfield J. (2024). Ketogenic diets are not beneficial for athletic performance: response to Noakes. Med. Sci. Sports Exerc..

[47] Burke L.M., Whitfield J. (2024). Ketogenic diets are not beneficial for athletic performance. Med. Sci. Sports Exerc..

[48] Fuller K.N.Z., Thyfault J.P. (2021). Barriers in translating preclinical rodent exercise metabolism findings to human health. J. Appl. Physiol..

[49] Stellingwerff T., Burke L.M., Caldwell H.G., Gathercole R.J., McNeil C.J., Napier C. (2025). Integrative field-based health and performance research: a narrative review on experimental methods and logistics to conduct competition and training camp studies in athletes. Sports Med.

[50] Burke L.M., Merrell L., Heikura I.A., Civil R., Sharma A.P., Leckey J.J. (2025). Caffeine enhances performance regardless of fueling strategy, however high CHO availability is associated with improved training speeds compared with ketogenic diet. Br. J. Nutr..

